# Different Inhibitory Potencies of Oseltamivir Carboxylate, Zanamivir, and Several Tannins on Bacterial and Viral Neuraminidases as Assessed in a Cell-Free Fluorescence-Based Enzyme Inhibition Assay

**DOI:** 10.3390/molecules22111989

**Published:** 2017-11-17

**Authors:** Stefanie Quosdorf, Anja Schuetz, Herbert Kolodziej

**Affiliations:** 1 Institute of Pharmacy, Freie Universität Berlin, Königin-Luise-Str. 2+4, 14195 Berlin, Germany; stquosdorf@gmail.com; 2 Max-Delbrück-Centrum for Molecular Medicine, Helmholtz Protein Sample Production Facility, Robert-Rössle-Str. 10, 13125 Berlin, Germany; anja.schuetz@mdc-berlin.de

**Keywords:** neuraminidase, inhibition, tannins, oseltamivir carboxylate, zanamivir, crystal structure, molecular interactions

## Abstract

Neuraminidase is a key enzyme in the life cycle of influenza viruses and is present in some bacterial pathogens. We here assess the inhibitory potency of plant tannins versus clinically used inhibitors on both a viral and a bacterial model neuraminidase by applying the 2′-(4-methylumbelliferyl)-α-d-*N*-acetylneuraminic acid (MUNANA)-based activity assay. A range of flavan-3-ols, ellagitannins and chemically defined proanthocyanidin fractions was evaluated in comparison to oseltamivir carboxylate and zanamivir for their inhibitory activities against viral influenza A (H1N1) and bacterial *Vibrio cholerae* neuraminidase (VCNA). Compared to the positive controls, all tested polyphenols displayed a weak inhibition of the viral enzyme but similar or even higher potency on the bacterial neuraminidase. Structure–activity relationship analyses revealed the presence of galloyl groups and the hydroxylation pattern of the flavan skeleton to be crucial for inhibitory activity. The combination of zanamivir and EPs^®^ 7630 (root extract of *Pelargonium sidoides*) showed synergistic inhibitory effects on the bacterial neuraminidase. Co-crystal structures of VCNA with oseltamivir carboxylate and zanamivir provided insight into bacterial versus viral enzyme-inhibitor interactions. The current data clearly indicate that inhibitor potency strongly depends on the biological origin of the enzyme and that results are not readily transferable. The therapeutic relevance of our findings is briefly discussed.

## 1. Introduction

Influenza is an acute viral infection of the respiratory tract, afflicting millions of individuals each year. Due to the imminent threat of epidemics and pandemics in human population and the alarming emergence of drug-resistant influenza virus strains, there is an urgent need for new and effective anti-influenza drugs. 

The influenza virus employs two key enzymes located on the surface, hemagglutinin (HA) and neuraminidase (NA), to initiate viral fusion and subsequent budding of progeny virions from the infected cell [1]. In the virus infection life cycle, HA binds to terminally linked sialic acid receptors on target cells, thus facilitating entry of the virus into the host cell. NA plays a key role not only in the release of virions from infected host cells by cleaving terminal sialic acid residues but also in preventing self-aggregation of the released influenza viruses, thereby allowing continuous and efficient viral replication.

Annual vaccination has been the main global strategy for preventing influenza infections, but frequent virus antigenic drifts present a permanent challenge in the development of effective vaccines. Antiviral drugs offer an alternative therapeutic option. Currently, there are two classes of anti-influenza drugs available, matrix-2 (M2) protein ion channel blockers and neuraminidase inhibitors (NAIs). The therapeutic efficacy of M2 ion channel blockers such as amantadine and rimantadine is limited to influenza A viruses. These therapeutics are also associated with the rapid emergence of drug resistance and have the disadvantage of side effects. At present, the approved NAIs oseltamivir, zanamivir, peramivir, and laninamivir are first choice antiviral drugs for the treatment of influenza A and B virus infections. Until the season 2007/2008, NAI-resistance to the first introduced NA-targeting drugs has been reported only sporadically [2]. Over the years, the emergence of oseltamivir-resistant strains is a cause for concern. Nevertheless, NAIs are currently considered to be the most promising drugs for combating influenza A and B. Despite their considerable therapeutic potential, the number of approved competitive inhibitors of the influenza NA is rather limited to date. Taking into account the serious problem of resistance development from antigenic shifts or drifts, there is a persistent demand to develop new NAIs.

A successful modus operandi for finding promising antiviral agents involves the exploration of plants and their chemical constituents. The plant kingdom has been recognized as an inestimably rich source of metabolites for target-oriented drug discovery. In particular, various plant polyphenols that have a broad spectrum of other biological activities have been identified to possess viral NA inhibitory activities [3,4,5]. Although isolated pure products have been preferentially elucidated for this bioactivity, some plant extracts, in particular traditional Chinese medicines have been subjected to anti-influenza activity studies [6,7,8,9]. 

NAs are also present in other biological systems [10], for example bacteria, and these non-influenza NAs are critical virulence factors in many pathogenic organisms including *Streptococcus pneumoniae*, *Pseudomonas aeruginosa*, and *Vibrio cholerae* [5,11,12]. Bacterial sialidases have been suggested to promote microbial survival and to contribute to microbe–host interactions [13]. Their function in pathogenesis remains to be clarified. However, bacterial NA activity has been shown to contribute to respiratory tract infections in a mouse model [14]. Taking into account the demonstrated synergism between influenza virus and bacterial pathogens in pulmonary infectious conditions resulting from the exposure of pneumococcal receptors [15], bacterial NAs may be an appealing target to prevent microbial colonization.

Established functional assays to assess NA inhibitory activities are based on either fluorescence or chemiluminescence and are reported to be compared to culture-based assays, more predictive in terms of their in vivo susceptibility [3]. The in vitro NA inhibition assays work with both viral and bacterial NAs, because the enzymes recognize the applied substrates independent of their biological origin [16]. The use of commercially available bacterial or viral NA-based test systems may be beneficial for a given pathogenic condition. In addition, bacterial NA-based setups are less expensive and are used for antiviral activity studies in laboratories. To date, however, comparative studies on the effect of tannins on bacterial and viral NAs are lacking. These comparisons could allow for the evaluation of the therapeutic significance of bacterial enzyme inhibition data in the identification of anti-viral substances [3].

To fill this gap, we here disclose results for the inhibition of both a viral and a bacterial model NA by various tannins. In addition to a range of flavan-3-ols and ellagitannins, highly purified and chemically defined proanthocyanidin fractions of different composition were included to gain insight into structure–activity relationships for this group of polyphenols. To provide a rationale for differential inhibition of bacterial and viral NAs, we additionally performed X-ray crystallographic analyses of NAs in complex with the synthetic reference compounds oseltamivir carboxylate and zanamivir.

## 2. Results

**Figure 1:**
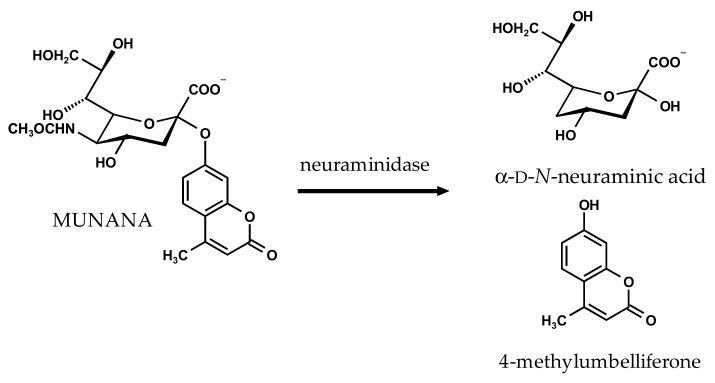
Neuraminidase-catalyzed hydrolysis of 2′-(4-methylumbelliferyl)-α-d-*N*-acetylneuraminic acid (MUNANA).

Preliminary experiments in our research group have indicated differential inhibition of bacterial and viral NAs by some polyphenolic substances [17] and have prompted the present more detailed study. We here explore the structure–activity relationship of a range of polyphenolic NAIs using the well-established 2′-(4-methylumbelliferyl)-α-d-*N*-acetylneuraminic acid (MUNANA)-based activity assay [18]. The assay is based on the NA-catalyzed hydrolysis of the substrate MUNANA and quantification of the released fluorochrome 4-methylumbelliferone (Figure 1). The established NAIs oseltamivir carboxylate (active form of oseltamivir) and zanamivir were included as reference compounds.

### 2.1. Inhibition of Viral Influenza A Neuraminidase and Bacterial Vibrio cholerae Neuraminidase by the Reference Compounds

**Figure 2:**
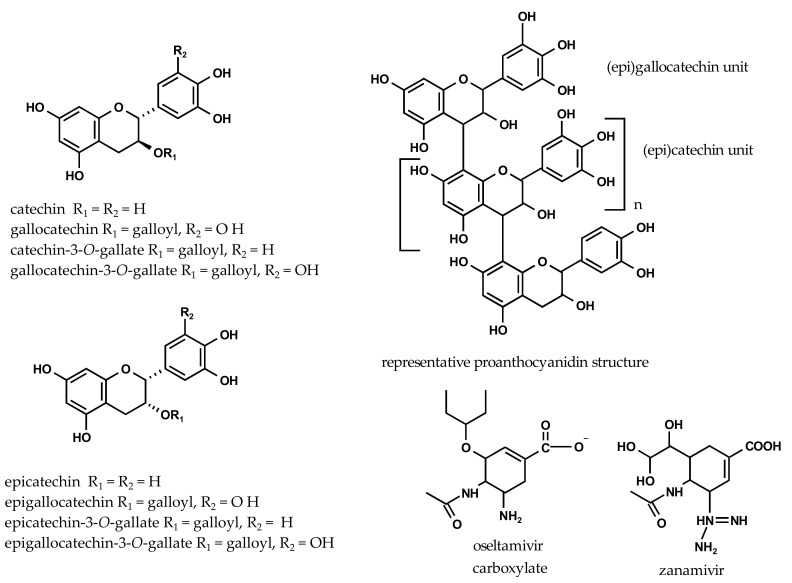
Chemical structures of tested flavan-3-ols, proanthocyanidins, oseltamivir carboxylate, and zanamivir.

To validate our assay conditions, we first assessed the inhibitory potency of the reference NAIs oseltamivir carboxylate and zanamivir (Figure 2) against both the viral influenza A/California/04/2009 (H1N1) neuraminidase (H1N1-NA) and the bacterial *Vibrio cholerae* neuraminidase (VCNA). With IC_50_ values (the inhibitor concentration that is required for 50% inhibition) of around 10 nM, oseltamivir carboxylate and zanamivir are very active against the viral H1N1-NA. The bacterial VCNA, on the other hand, is only moderately inhibited by the reference compounds, displaying IC_50_ values of 144 µM for oseltamivir carboxylate and 52 µM for zanamivir. These IC_50_ values are comparable to other NA inhibition data [19,20], although modified assay conditions were used.

### 2.2. Inhibition of Viral H1N1-NA and Bacterial VCNA by Flavan-3-ols

The inhibitory potencies of a series of flavan-3-ols are shown in Table 1, revealing markedly different inhibitory activities towards the bacterial and viral NAs. All tested flavan-3-ols (Figure 2) display only moderate to very weak inhibitory activities against the viral H1N1-NA when compared to the synthetic reference inhibitors. The IC_50_ values range from 0.3 to 0.9 mM, and gallocatechin-3-*O*-gallate is the most active compound within this series. This finding is consistent with modest inhibitory activities (>100 µM) reported for (*epi*)catechin [3].

In contrast, these compounds are effective inhibitors of the bacterial VCNA. The flavan-3-ols gallocatechin-3-*O*-gallate (IC_50_ = 25 µM), catechin-3-*O*-gallate (IC_50_ = 55 µM), and epigallocatechin-3-*O*-gallate (IC_50_ = 64 µM) have comparable or greater potency compared to the reference compounds (Table 1). All tested non-galloylated flavan-3-ols, on the other hand, show comparatively weak inhibitory activities, indicating that a 3-*O*-galloyl group is crucial for pronounced inhibition of VCNA by polyphenols. The enhanced inhibitory activity of the 2,3-*trans* galloylated flavan-3-ols compared with their 2,3-*cis* analogues suggests that the relative 2,3-configuration is, to some extent, an additional structural feature contributing to the inhibition of the bacterial NA.

### 2.3. Inhibition of Viral H1N1-NA and Bacterial VCNA by Ellagitannins

**Figure 3:**
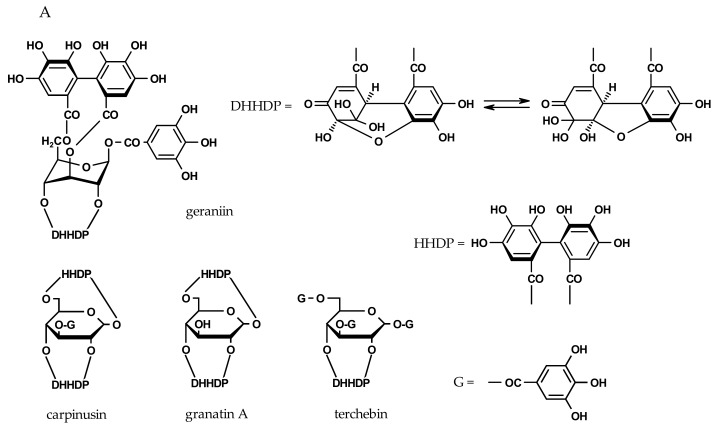

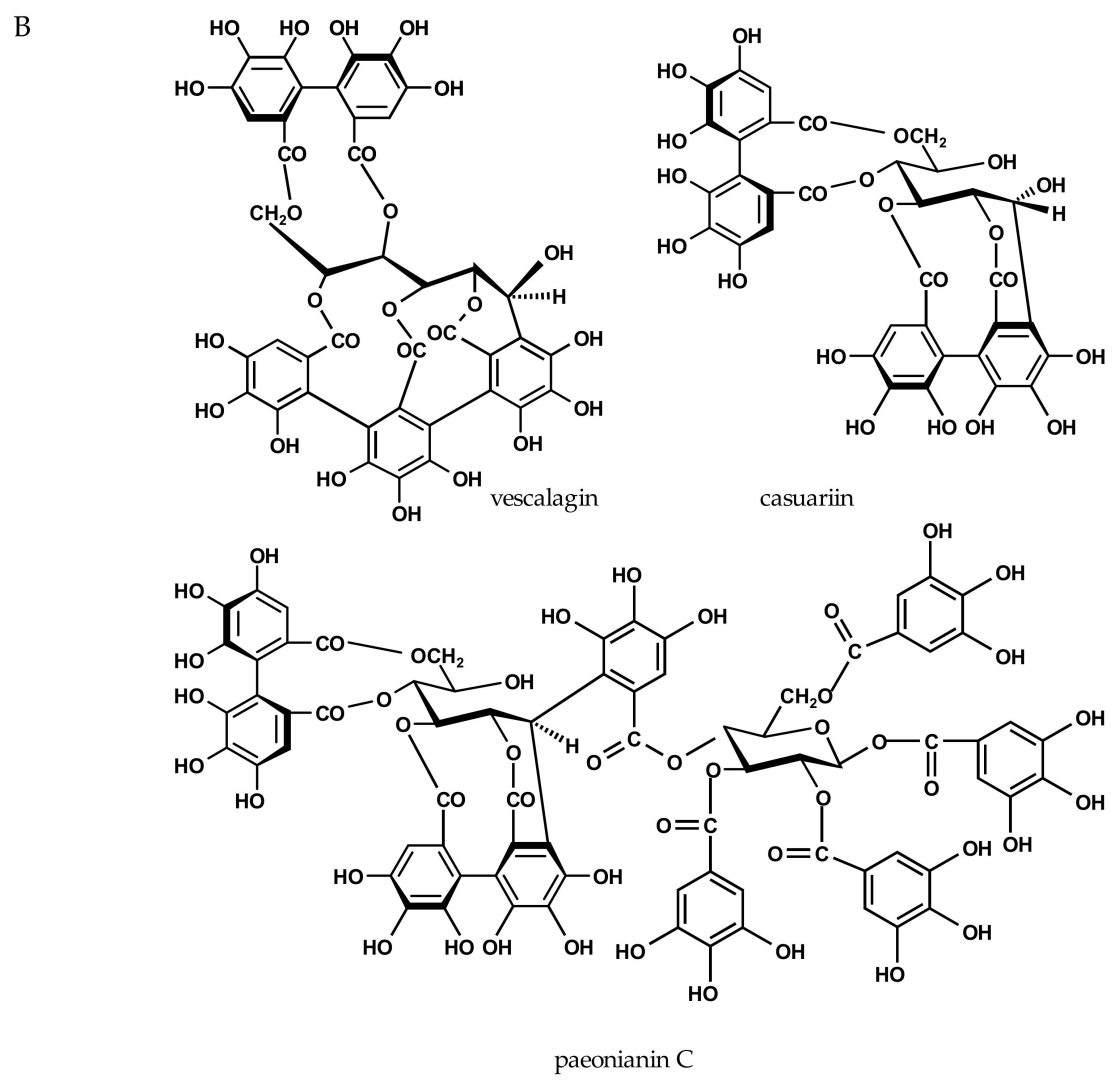
Chemical structures of ellagitannins. (**A**) Dehydroellagitannins and (**B**) *C*-glycosidic members (DHHDP = dehydrohexahydroxydiphenoyl; HHDP = hexahydroxydiphenoyl).

We next evaluated the inhibitory potency of a series of ellagitannins (Figure 3), including members of dehydroellagitannins and *C*-glycosidic ellagitannins. All ellagitannins inhibit bacterial VCNA with an IC_50_ value of 15–236 µM. The most potent compounds, which were even more effective than zanamivir, are paeonianin C (IC_50_ = 15 µM) and terchebin (IC_50_ = 31 µM). In tests with the viral H1N1-NA, the inhibition is also in the µM range, but terchebin showed a 3.5-fold higher inhibitory potency compared to paeonianin C (Table 2). Overall, the ellagitannins are less effective against the viral compared to the bacterial NA. It is also worth mentioning that the only moderately effective VCNA ellagitannin inhibitors are still as potent as the reference compound oseltamivir carboxylate.

A structural element characteristic of dehydroellagitannins is the presence of at least one dehydrohexahydroxydiphenoyl (DHHDP) unit in addition to a varying number of galloyl groups on the glucose core. Structure–activity relationship analyses indicate that the inhibitory activity of the tested compounds depends in part on the degree of galloylation. As shown in Table 2, inhibition decreased in the order of terchebin > geraniin > carpinusin > granatin A (IC_50_ values of 31, 135, 138 and 158 µM), corresponding to three, one, and no galloyl groups. Further examination of the structures revealed that the presence of additional 1′,6′- or 3′,6′-hexahydroxydiphenoyl (HHDP) residue (Figure 3) results in significantly weaker inhibitory activities, possibly due to steric effects.

Although the number of tested *C*-glycosidic ellagitannins was limited, some structural features determining inhibitory potency were evident. Paeonianin C, possessing a 1C-penta-*O*-galloyl glucose moiety, was significantly more potent (IC_50_ = 15 µM) than vescalagin (IC_50_ = 73 µM), having a 4,6-HHDP and a 2,3,5-flavogalloyl unit, and casuariin (IC_50_ = 236 µM), which lacks the 5-*O*-galloyl residue as part of the flavogalloyl group. The inhibition correlates with the number of pyrogallol elements. More information is needed to decide between galloyl-derived structural variants. However, it appears that the conformation of these molecules with bulky substituents plays a significant role and more *C*-glycosidic ellagitannins need to be tested to fully understand the structure–activity relationships.

### 2.4. Inhibition of Viral H1N1-NA and Bacterial VCNA by Plant-Derived Fractions

Proanthocyanidins are oligomeric flavan-3-ols and represent another group of plant tannins. Extensive structural variations and challenges in the isolation and purification of chemically defined proanthocyanidins prompted the evaluation of well-characterized plant fractions. Table 3 lists the main component of the composite fractions and the resulting IC_50_ values for both viral H1N1-NA and bacterial VCNA inhibition. Most of the tannin fractions inhibit the bacterial VCNA significantly better than the reference compounds. The inhibition of viral H1N1 is reduced by a factor of 6–10 compared to the clinically used inhibitors, leaving potential for further optimization.

For both NAs, the *Diospyros kaki* sample is the most active fraction, suggesting that pyrogallol B-ring elements (prodelphinidin units) and 3-*O*-galloylation are preferred structural determinants for NA inhibition. In line with this, weaker inhibitory activity is found for the prodelphinidin-rich but mainly ungalloylated extract of *Pelargonium sidoides* EPs^®^ 7630 extract. The trend continues for the *Nelia meyeri* and *Salix* spp. fractions, which comprise either homogeneous 2,3-*cis* or 2,3-*trans* proanthocyanidins composed of non-galloylated dimers to hexamers in similar proportions. Interestingly, fractions with mixed 2,3-*trans* and 2,3-*cis* constituent flavanyl units obtained from *Potentilla erecta* and *Betula* spp. are less potent than preparations dominated by compounds with the homogeneously linked building blocks. Increasing proportions of 2,3-*cis* entities as in the *Betula* fraction apparently reduces the inhibitory activity (Table 3). More studies with fractions of defined 2,3-*cis*/2,3-*trans* ratios and a focus on the impact of the molecular weight are fruitful avenues for future research.

The data additionally show the relatively poor activity of 5-deoxy analogues, such as the compounds in the *Rhus leptodictya* fraction. The profisetinidins with characteristic resorcinol A-rings show the weakest inhibition within the series of tested proanthocyanidin fractions. The hydroxylation pattern on the two aromatic rings of the flavan skeleton thus proves to be an important structural feature.

### 2.5. Combined Effect of Zanamivir and EPs^®^ 7630 on VCNA Activity

Possible beneficial effects of combination therapy include synergistic activity, reduced dosage with decrease of side effects, and delay or prevention of development of drug resistance. Indeed, previous reports have stated advantages of synthetic NAI combinations over single-drug influenza treatment [21,22,23,24]. Additionally, drug–herb combinations such as the Manuka honey constituent methylglyoxal along with oseltamivir [25] as well as also some entirely herbal-based combinations [26] proved to be effective anti-influenza NAIs.

Recent work indicates that bacterial NAs play a role in the pathogenesis of respiratory tract infections [14]. However, little information is available on natural products inhibiting bacterial NA activity. We thus examined the hitherto less studied drug–herb combination approach and determined the combined effect of zanamivir and EPs^®^ 7630 on bacterial VCNA using the median-plot effect analysis [27,28]. Selection of EPs^®^ 7630 was based on its VCNA-inhibition potential demonstrated in this study and its approval for the treatment of acute bronchitis [29]. The clinically used drug zanamivir was chosen because it is more effective against VCNA than oseltamivir carboxylate against VCNA.

The enzyme was treated with zanamivir and EPs^®^ 7630 either individually over a range of concentrations or in combination at different weight ratios (1:10, 1:5, 1:1, 5:1, or 10:1) (Table 4). The median–effect concentration (*D_m_*, a parameter analogous to the IC_50_ value indicating potency), the slope of the concentration–effect relationship (*m*, the dynamic order or the shape of dose–effect curve), and the linear regression correlation coefficient (*r*, a measure of data quality) were determined from the median–effect plots. The parameter *r* of the median–effect plots exceeds 0.95 (data not shown), showing the conformity of the data to the median–effect principle. The calculated *D_m_* values indicate an enhanced VCNA inhibition with increasing amounts of EPs^®^ 7630 (ratio of 1:5 and 1:10, *D_m_* = 1.4) as compared to zanamivir alone (*D_m_* = 17). 

**Figure 4:**
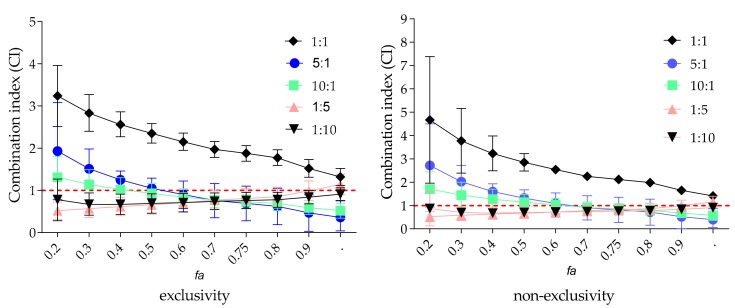
Fraction affected (*f_a_*)–CI-plot for VCNA activity measurements by zanamivir/EPs^®^ 7630-combinations. The vertical bars indicate 95% confidence intervals (*n* = 3–4 independent experiments).

We calculated the combination index (CI) values that offer a quantitative definition for an additive effect (CI = 1), synergism (CI < 1), and antagonism (CI > 1) in drug combinations [27,28]. CI values were determined at IC_50_, IC_75_, IC_90_, and IC_95_, indicating the concentrations required to achieve 50%, 75%, 90% or 95% inhibition of VCNA activity. It should be noted that only CIs of higher effect levels (fraction affected values, *f_a_* > 0.5) are considered relevant for therapeutic applications [30]. The *f_a_*-CI plots for various zanamivir/EPs^®^ 7630 combinations are shown in Figure 4. The CI values are concentration-dependent and are generally higher with lower sample concentrations. The tendency of combined effects moving from antagonism towards additivity and synergism as effect levels increase, appears to be a common phenomenon. Indeed, antagonism is restricted to the region of lower *f_a_* values in our study. For higher effect levels, CI values move to synergism except for the 1:1 combination, exhibiting continuously antagonistic effects (Figure 4). The combined effect (CI_wt_) was calculated on the basis of selected average CI values including IC_50_, IC_75_, IC_90_ and IC_95_ at four representative effect levels (*f_a_* > 0.5). The most effective combinations include drug–herb ratios of 10:1, 5:1, and, surprisingly, 1:10, with CIs ranging between 0.5 and 0.8, indicative of synergism. For the zanamivir/EPs^®^ 7630 combination with a ratio of 1:5, the CI revealed additivity. 

Differences in the slopes of the median effect plots for EPs^®^ 7630 (*m* > 1, sigmoidal) and zanamivir (*m* < 1, flat sigmoidal) alone and for the tested combinations (all *m* > 1) indicate possible different binding sites for each compound. Therefore, the data were reanalyzed with an assumption of non-exclusivity for the calculations of CI values of the tested combinations (Table S1). However, the overall combined effects (CI_wt_) are very similar for all the tested combinations, whether the calculation was based on exclusivity or non-exclusivity. Further analyses of the complex EPs^®^ 7630 extract composition [29,31] might be beneficial, but are beyond the scope of this work.

### 2.6. Molecular Basis of NA-Inhibitor Interactions as Assessed by Crystallographic Analyses

The in vitro studies presented here unambiguously show the different inhibitory potency of tannins against bacterial and viral NAs. To better understand the differential NA–tannin interactions at the molecular level and to assess the specificity of tannin binding, we aimed at solving the crystal structures of various NA–flavan-3-ol complexes. These attempts, however, remained unsuccessful. Instead, we solved the crystal structures of bacterial VCNA in complex with the reference compounds oseltamivir carboxylate and zanamivir at 1.87 and 1.75 Å resolution (Figure S1), allowing a comparative analysis of bacterial versus viral enzyme-inhibitor interactions for these synthetic substances. Data collection and refinement statistics are reported in Table 5.

The uncomplexed crystal structures of influenza A H1N1-NA and bacterial VCNA have been previously published. The enzymes have different overall structural topologies. The viral enzyme is a mushroom-like shaped homotetramer, while the bacterial NA is a monomer with two flanking lectin-like domains [32,33,34]. Both enzymes share the canonical six-blade β-propeller fold of the catalytic NA domain, despite exhibiting a relatively low amino acid sequence identity of only 12% as revealed by structure-based sequence alignment (Figure S2). Co-crystal structures of H1N1-NA in complex with zanamivir and oseltamivir carboxylate are publicly available (Protein Data Bank (PDB) ID codes 3TI5 and 3TI6) [19], and were used for the following detailed comparison of protein–ligand interactions with the herein determined new bacterial VCNA complex structures.

**Figure 5:**
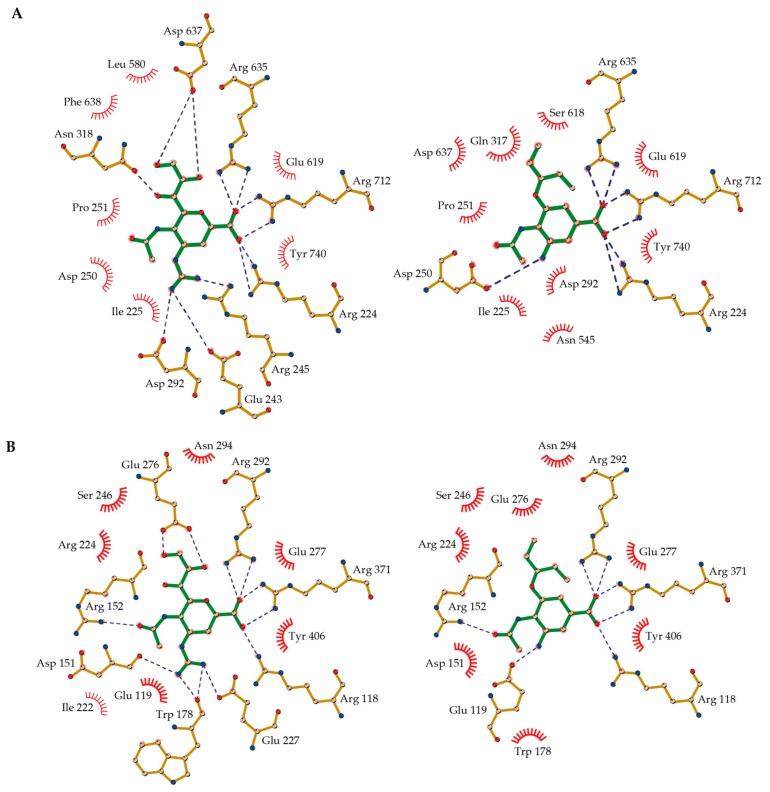
Binding modes of zanamivir (left, green stick model) and oseltamivir carboxylate (right, green stick model) in the active site of (**A**) VCNA, determined in this study (Protein Data Bank (PDB) codes 6EKU and 6EKS); and (**B**) H1N1-NA (PDB codes 3TI5 and 3TI6) [19]. Hydrogen bonds are depicted with dashed blue lines and hydrophobic interactions are shown as red arcs. The figures were prepared with LigPlot^+^ [36].

A common feature of viral and bacterial inhibitor binding is the electrostatic and hydrogen bonding interaction of the carboxylic acid group of oseltamivir carboxylate or zanamivir to a cluster of three positionally conserved arginine residues (Arg224, Arg635, and Arg712 in VCNA; Arg118, Arg292, and Arg371 in H1N1-NA) (Figure 5, Tables S2 and S3). Notably, Arg224 of VCNA belongs to the bacterially conserved RIP/RLP motif and Arg118 of H1N1-NA belongs to the corresponding REP motif of viral NAs [35]. A second common structural feature is the electrostatic and hydrogen bonding interaction of the 5-amino group of oseltamivir carboxylate or the 4-guanidino moiety of zanamivir with an electronegative binding pocket within the active site (Glu243, Arg 245, Asp250 and Asp292 in VCNA; Glu119, Asp151, and Glu227 in H1N1-NA) (Figure 5, Tables S2 and S3). Notable differences between viral and bacterial inhibitor complexes are observed in the surrounding region of the acetamido moiety of oseltamivir carboxylate and zanamivir. In viral H1N1-NA, the side chain of Arg152 is hydrogen bonded to the acetamido moiety of the inhibitors, an interaction that is missing in the bacterial complexes (Figure 5). Moreover, the acetamido binding cavity in bacterial VCNA is sterically constrained by the short amino acid stretch of Gln317 and Asn318 that extends into the active site. Another steric restriction in the bacterial VCNA active site is seen in the microenvironment around the 5-amino group of oseltamivir carboxylate and the 4-guanidino moiety of zanamivir. Glu243 and Arg245 extend further into the active site compared to Leu134 and Arg156 of the influenza enzyme, thereby reducing the available space in that subpocket. Thus, oseltamivir carboxylate and zanamivir fit much better into the active site of influenza H1N1-NA than that of bacterial VCNA, consistent with their relative inhibitory potencies (Table 1). Taken together, the structural differences between H1N1-NA and VCNA inhibitor complexes contribute to the observed differential inhibition of viral and bacterial NA by oseltamivir carboxylate and zanamivir.

To rationalize the observed increased inhibitory activity of zanamivir over oseltamivir carboxylate for bacterial VCNA (IC_50_ of 52 µM versus 144 µM, Table 1), we compared the inhibitor interactions in the determined VCNA complex structures. The interaction network differs most in those regions where the chemical structure of the inhibitors is different. The 4-guanidino group of zanamivir, for example, forms electrostatic and hydrogen bonding interactions with Glu243, Arg245, and Asp292, whereas the 5-amino group of oseltamivir carboxylate interacts with Arg250 only (Figure 5, Table S3). The hydrophilic 6-(1′,2′,3′)-trihydroxypropyl group of zanamivir hydrogen bonds with Asn318 and Asp637, the corresponding 3-(1′-ethylpropoxy) group of oseltamivir carboxylate, however, interacts only hydrophobically. Furthermore, the oxygen within the 5,6-dihydro-4*H*-pyran ring of zanamivir forms a hydrogen bond with the side chain of Tyr740, while the cyclohex-1-ene entity of oseltamivir carboxylate interacts exclusively hydrophobically (Figure 5, Table S3). Summarizing, the elevated number of hydrogen bonds in the zanamivir complex likely explains the increased potency of zanamivir over oseltamivir carboxylate for bacterial VCNA inhibition.

## 3. Discussion

The current data clearly indicate a differential inhibition of the viral H1N1-NA and bacterial VCNA by the various tested tannins and the reference compounds. While oseltamivir carboxylate and zanamivir showed pronounced inhibition of the viral H1N1-NA, the polyphenolic substances were more effective against bacterial VCNA. Thus, IC_50_ values obtained from a fluorometric activity assay using neuraminidases of different biological origin are not readily comparable and it is therefore advisable to select viral NAs when screening compounds for their anti-influenza potencies, and bacterial NAs when the testing is aimed at targeting bacterial pathogens.

Previous structure–activity relationship analyses of various groups of flavonoids revealed that the presence of the 4-keto function and the C2–C3 double bond are relevant structural elements for potent viral NA inhibition [3,37]. These chemical features are “missing” in flavan-type molecules such as the flavan-3-ols tested in this study, consistent with their weak viral inhibitory activity. In contrast, oligomeric flavan-3-ols (proanthocyanidins) show more promise as antiviral candidates. A computational virtual screening recorded proanthocyanidins within the top ranked plant metabolites selected from ligand databases, but detailed chemical information is lacking [38]. We demonstrate that pure fractions of proanthocyanidins are only 6–10 times less potent compared with the viral-specific reference NA inhibitors. Given the bioactive potential of proanthocyanidins, compositional extract optimization and structural modifications may improve their ability to inhibit viral NAs. Proanthocyanidins may furthermore be sufficiently active against influenza by different modes of action. Targets other than NAs could well provide more potent antiviral effects as exemplarily reported for EPs^®^ 7630 [39].

Next to possible antiviral effects, tannins may also be beneficial for treating bacterial infections. Importantly, NAs of many bacterial, respiratory pathogens play a key role in early stages of pulmonary infection by forming biofilms that contribute to initial colonization of the respiratory tract [14]. Bacterial NAs may additionally be involved in the pathogenesis of enteric bacterial infections [40]. Modulation of bacterial NAs by, for example, secondary plant products thus presents an appealing approach to prevent bacterial infection of the respiratory tract and to maintain gut homeostasis. We analyzed the inhibitory potential of a series of flavan-3-ols and chemically defined proanthocyanidin fractions on the bacterial NA from *Vibrio cholerae*. Compared to the reference NAIs, the proanthocyanidin samples are significantly more potent. We demonstrate that the hydroxylation pattern of the flavan skeleton of the inhibitors and the presence of a 3-*O*-galloyl group is a key structural feature for effective VCNA inhibition. Moreover, inhibitor potency is correlated with the number of molecular interactions within the active site as shown by our crystal structure analysis of VCNA complexes with zanamivir and oseltamivir carboxylate. Within the series of ellagitannins, paeonianin C and terchebin show superior VCNA inhibitory activities compared to zanamivir and oseltamivir carboxylate, a finding that deserves further attention. Overall, tannins exhibit remarkable VCNA inhibitory potencies, a stimulus for similar studies on other bacterial NAs.

The impact of oseltamivir carboxylate and zanamivir on the activity of other bacterial NAs has been reported in previous publications [41,42,43]. The *Streptococcus pneumoniae* NA NanA, for example, has a K_i_ value of 1.77 μM for oseltamivir carboxylate and 0.72 mM for zanamivir [41]. Also the *Ruminococcus gnavus* NA is more effectively inhibited by oseltamivir carboxylate than by zanamivir (IC_50_ of 30 µM versus 11.89 mM) [42]. In contrast, we observed an increased inhibitory activity of zanamivir over oseltamivir carboxylate for bacterial VCNA (IC_50_ of 52 µM versus 144 µM, Table 1), consistent with recent findings [44]. This highlights the difficulties in translating structure-based drug developments even between bacterial NAs of different origin, though NAs share a similar fold in their active sites [43]. 

Another important finding of our study is that the drug–herb combination zanamivir and EPs^®^ 7630 is a more potent inhibitor of bacterial VCNA than either compound alone, suggesting promising benefits of similar combinatory therapies. Clinical studies are needed to approve the therapeutic potential. The fact that herbal medicines are increasingly used as an adjuvant treatment of both bacterial and viral infections makes this field a promising but challenging research in the future.

Finally, absorption, distribution in tissues, and metabolism are important efficacy-influencing parameters. The intestine and its microbiota play a crucial role in this process. Further research is needed to clarify the impact of microbial degradation of tannins and their metabolites on health effects.

## 4. Materials and Methods 

### 4.1. Chemicals

4-Methylumbelliferone, 2′-(4-methylumbelliferyl)-α-d-*N*-acetylneuraminic acid (MUNANA), and 2-(*N*-morpholino) ethanesulfonic acid (MES) were purchased from Sigma Aldrich (Taufkirchen Germany). Sources of NAIs: Oseltamivir carboxylate (5-*N*-acetyl-3-(1-ethylpropyl-1-cyclohexene-1-carboxylic acid), the active form of oseltamivir, was kindly supplied by Hoffmann La Roche (Basel, Switzerland) and zanamivir (2,4-dideoxy-2,3-didehydro-4-guanidino-*N*-acetylneuraminic acid) by GlaxoSmithKline (Brentford, Middlesex, UK). All flavan-3-ols (purity > 95%) were commercially obtained (catechin, Roth, Germany; gallocatechin and catechin-3-*O*-gallate, Sigma Aldrich, Taufkirchen, Germany; gallocatechin-3-*O*-gallate and epicatechin, Fluka, Switzerland; epigallocatechin, Alfa Aesar, Germany; epicatechin-3-*O*-gallate, AppliChem, Darmstadt, Germany; epigallocatechin-3-*O*-gallate, Tocris Bioscience, Bristol, UK), while the ellagitannins are generous gifts from Prof. T. Yoshida, Okayama University, Japan. The highly purified proanthocyanidin fractions obtained from *Nelia meyeri*, *Salix* spp., *Betula* spp., *Potentilla erecta*, *Rhus leptodictya*, and *Diospyros kaki* were available in our research group. The preparation and characterization of the oligomeric proanthocyanidin mixtures are described elsewhere [45,46,47,48,49,50]. The root extract of *Pelargonium sidoides* (EPs^®^ 7630, an aqueous-ethanolic extract), was obtained from Dr. Willmar Schwabe Pharmaceuticals (Karlsruhe, Germany).

### 4.2. NA Inhibition Assay

To assess NA activity, a fluorometric NA inhibition assay using MUNANA as a substrate and commercially available *Vibrio cholerae* NA was performed [18]. VCNA (Hoffmann La Roche, Basel, Switzerland) (1 U stock) was diluted 1:10 in MES-buffer (34.8 µM MES-NaOH pH 6.5, 4 mM CaCl_2_) and stored at −22 °C in aliquots, while the viral NA (influenza virus strain/California/04/2009; H1N1; BioTrend, Köln, Germany) in Tris-buffer (20 mM Tris-HCl pH 7.4, 500 mM NaCl, 10% glycine) was adjusted to 80–120 mU/mL with distilled water and kept in aliquots at −80 °C until use. Stock solutions (0.1–10 mg/mL) of the test substances were prepared in MES-buffer, aqueous ethanol or DMSO/H_2_O (1:5 *v*/*v*) and serially diluted in MES-buffer for measurements.

To quantify enzyme activity, 60 µL of MES-buffer and 10 µL of diluted VCNA were mixed with 10 µL of varying concentrations of the test substance in black 96-well plates. For analyses of the viral NA, half of the volumes were used. After agitating gently and incubating for 2 min at 37 °C, 20 µL of the substrate MUNANA (1 mM stock solution) was added, and the fluorescence measured over the course of 14 min at an excitation wavelength of 360 nm and an emission wavelength of 465 nm. Appropriate controls were included in the measurements. All assays were at least performed in triplicates (*n* = 3–6) and IC_50_ values for each substance were calculated from fitting curves to data points (percentage of inhibition vs. concentration) using GraphPad Prism version 6.0 for Windows (GraphPad Software, La Jolla, CA, USA, www.graphpad.com). 

IC_50_ values are expressed as mean values ± standard deviation for *n* = 3–6 experiments. For normally distributed data, first the variance homogeneity test (F-test) was applied, followed by the *t*-test. For data with non-normal distributions, the Welch-modified *t*-test was used. Analysis of variance (ANOVA) followed by the Bonferonni’s multiple comparison test was used to evaluate differences between tests. *p*-values <0.05 were considered statistically significant.

### 4.3. Drug Combination Analysis

The combined effect of zanamivir and EPs^®^ 7630 on VCNA was determined using the diagonal constant ratio combination method designed by Chou and Talalay [27]. VCNA was incubated with zanamivir or EPs^®^ 7630 individually and in combination at weight ratios of zanamivir to EPs^®^ 7630 (10:1, 5:1, 1:1, 1:5, and 1:10) as described in Section 4.2. The concentration–efficiency curves of the test samples were transformed into the double-logarithmic mean action diagram (shape (*m*), intercepts and regression coefficients were used to determine the median effect dose *D*_m_).

$$D_{m}=\;10^{-\left(\frac{b}{m}\right)}$$

Fraction affected (*f_a_*) and fraction unaffected (*f_u_*) by the concentration *D* were calculated by the median effect equation [27]:

$$\frac{f_{a}}{f_{u}}=\;{\left(\frac{D}{D_{m}}\right)}^{m}$$

*D_m_* is the concentration required to produce the median effect.

The CI for quantification of synergism, additive effects, or antagonism was calculated as follows:

(1) $$\mathrm{CI}=\frac{D_{1}}{{\left(D_{x}\right)}_{1}}+\frac{D_{2}}{{\left(D_{x}\right)}_{2}}\;\left(\mathrm{exclusivity}\right)$$

(2) $$\mathrm{CI}=\frac{D_{1}}{{\left(D_{x}\right)}_{1}}+\frac{D_{2}}{{\left(D_{x}\right)}_{2}}+\frac{D_{1}\times \;D_{2}}{{\left(D_{x}\right)}_{1}\times \;{\left(D_{x}\right)}_{2}}\;\left(\mathrm{non}-\mathrm{exclusivity}\right)$$

Due to the therapeutic significance of the high degrees of effects, the weighted average CI values were calculated as

(3) $${\mathrm{CI}}_{\mathrm{wt}}=\;\frac{{\mathrm{CI}}_{50}\;+\;2\times {\mathrm{CI}}_{75}\;+\;3\times {\mathrm{CI}}_{90}\;+\;4\times {\mathrm{CI}}_{95}}{10}$$

### 4.4. Expression and Purification of Recombinant VCNA

The expression vector pET30b(+) that produces *Vibrio cholerae* serotype O1 (strain ATCC 39315/E1 Tor Inaba N165961) NA was a gift from Prof. G. Taylor, University of St. Andrews, Scotland. VCNA was produced according to a previously reported method with the following modifications [51]. VCNA was expressed in *E. coli* Rosetta™ 2 (DE3) (Merck KGaA, Darmstadt, Germany) using an LEX ultra-high-throughput bench-top bioreactor (Epiphyte3 Inc., Toronto, ON, Canada). Cells were grown at 37 °C in Terrific Broth medium to an optical density at 600 nm (OD_600_) of about 2.0–2.5, cooled to 17 °C, and induced with 0.5 mM isopropyl β-d-1-thiogalactopyranoside (IPTG). Cultures were then left shaking overnight, and cells were collected by centrifugation. 

For purification, cells were resuspended in 20 mM Tris–HCl pH 7.6 supplemented with cOmplete™ ethylenediamine tetra-acetic acid (EDTA)-free protease inhibitor cocktail (Merck KGaA, Darmstadt, Germany), and lysed by sonication (SONOPULS HD 2200, Bandelin Electronic GmbH & Co. KG, Berlin, Germany). The purification procedure comprised initial ammonium sulfate precipitation (50% *w*/*v*), desalting on a 26/10 HiPrep Desalting column (GE Healthcare, Munich, Germany) equilibrated with 10 mM Tris-HCl pH 7.6 and 150 mM NaCl, a Source 30Q anion exchange column, and a final size-exclusion chromatography on a Superdex 200 prep grade column (XK 26 × 60, GE Healthcare, Munich, Germany). Purified protein was concentrated to about 6 mg/mL in a buffer containing 20 mM Tris-HCl, pH 7.6, 0.15 M NaCl, and 10 mM CaCl_2_.

### 4.5. VCNA Crystallization and Structure Determination

The VCNA-inhibitor complexes were crystallized using the sitting-drop vapor-diffusion method at 20 °C by mixing equal volumes (200 nL) of purified protein (see Section 4.4) and reservoir solution, with a reservoir volume of 75 µL in 96-well plates. VCNA (in 20 mM Tris-HCl pH 7.6, 0.15 M NaCl, 10 mM CaCl_2_) was complexed with a 20 mM inhibitor (oseltamivir, zanamivir) and concentrated to 6–7 mg/mL. The VCNA–oseltamivir complex was crystallized using 20% (*w*/*v*) polyethylene glycol (PEG) 3350 and 0.2 M lithium acetate as reservoir solution. For the VCNA–zanamivir complex, the reservoir solution contained 20% (*w*/*v*) PEG 3350 and 0.2 M sodium fluoride. Before flash-freezing in liquid nitrogen, the crystals were transferred into a cryoprotectant consisting of reservoir solution supplemented with 30% (*v*/*v*) glycerol. 

X-ray diffraction data were collected at beamline BL14.1 at the Helmholtz-Zentrum Berlin [52] at a wavelength of 0.9184 Å and a temperature of 100 K. Data were processed with the program Xdsapp [53]. The structure was solved by molecular replacement using the program Phaser [54] and uncomplexed VCNA as search model (PDB entry 1KIT) [34]. Structure refinement was done with the Phenix program suite [55]. The graphics program Coot was used for manual model building and visualization [56]. Prodrg was used for generation of ligand restraint parameters [57]. Data collection and refinement statistics are reported in Table 5. Coordinates and structure factors for the VCNA-inhibitor complex structures have been deposited in the Protein Data Bank under accession codes 6EKS and 6EKU.

### 4.6. Multiple Sequence Alignment

The structure-based sequence alignment was performed with PDBeFold [58] using the PDB entries 1W0O (VCNA) and 3NSS (H1N1-NA). The aligned amino-acid sequences were transferred to the Clustal Omega server [59] to generate the input file for TEXshade [60], which was used for final visualization. The Uniprot accession numbers for *Vibrio cholerae* NA and influenza virus H1N1 A/California/04/2009 NA are P0C6E9 and C3W5S3.

## Figures and Tables

**Table 1:** IC_50_ values for the inhibition of viral influenza A (H1N1) neuraminidase (H1N1-NA) and bacterial *Vibrio cholerae* neuraminidase (VCNA) by flavan-3-ols.

Test Substance	IC_50_ (Viral) µg/mL µM		IC_50_ (Bacterial) µg/mL µM	
**Positive control**				
oseltamivir carboxylate	2.9 ± 0.2 ^(1)^	0.01 ± 0.001	41 ± 1	144 ± 1
zanamivir	3.7 ± 0.4 ^(1)^	0.01 ± 0.001	17 ± 1	52 ± 2
**Flavan-3-ols**				
catechin	312 ± 21	1076 ± 75	595 ± 25	2050 ± 87
gallocatechin	547 ± 23	1787 ± 74	603 ± 61	1969 ± 199
catechin-3-*O*-gallate	862 ± 2	1949 ± 4	24 ± 2	55 ± 4
gallocatechin-3-*O*-gallate	181 ± 3	396 ± 7	11 ± 1	25 ± 2
epicatechin	305 ± 19	1053 ± 64	670 ± 29	2186 ± 99
epigallocatechin	532 ± 41	1739 ± 135	598 ± 57	1955 ± 185
epicatechin-3-*O*-gallate	845 ± 24	1910 ± 55	93 ± 8	211 ± 19
epigallocatechin-3-*O*-gallate	717 ± 63	1565 ± 137	29 ± 1	64 ± 3

IC_50_ values are expressed as mean ± standard deviation (SD) (*n* = 3–6 independent experiments); ^(1)^ data are in ng/mL.

**Table 2:** IC_50_ values for the inhibition of viral H1N1-NA and bacterial VCNA by ellagitannins.

Test Substance	IC_50_ (Viral) µg/mL µM		IC_50_ (Bacterial) µg/mL µM	
**Positive control**				
oseltamivir acid	2.9 ± 0.2 ^(1)^	0.01 ± 0.001	41 ± 1	144 ± 1
zanamivir	3.7 ± 0.4 ^(1)^	0.01 ± 0.001	17 ± 1	52 ± 2
**Ellagitannins**				
dehydroellagitannin members				
geraniin	-	-	128 ± 2	135 ± 2
granatin A	-	-	124 ± 2	158 ± 4
carpinusin	-	-	131 ± 5	138 ± 5
terchebin	97 ± 2	101 ± 3	29 ± 2	31 ± 2
*C*-glycosidic members				
casuariin	-	-	185 ± 4	236 ± 5
vescalagin	-	-	125 ± 11	73 ± 11
paeonianin C	587 ± 24	344 ± 14	25 ± 2	15 ± 1

IC_50_ values are expressed as mean ± SD (*n* = 3–6 independent experiments); ^(1)^ data are in ng/mL.

**Table 3:** IC_50_ values for the inhibition of viral H1N1-NA and bacterial VCNA by tannin fractions.

Tested Substance	Constituent Flavanyl Units	IC_50_ (Viral) µg/mL	IC_50_ (Bacterial) µg/mL
**Positive control**			
oseltamivir acid		2.9 ± 0.2 ^(1)^	41 ± 1
zanamivir		3.7 ± 0.4 ^(1)^	17 ± 1
**Tannin fractions**			
*Diospyros kaki*	galloylated flavan-3-ols	20 ± 1	0.5 ± 0.04
EPs^®^ 7630 ^(2)^	(*epi*)gallocatechin/(*epi*)catechin	61 ± 2	1.7 ± 0.1
*Nelia meyeri*	epicatechin	29 ± 1	3.2 ± 0.1
*Salix* ssp.	catechin	32 ± 3	4.4 ± 0.2
*Potentilla erecta*	epicatechin/catechin	-	9.2 ± 1
*Betula* spp.	epicatechin/catechin	-	13 ± 1
*Rhus leptodictya*	fisetinidol	-	25 ± 1

IC_50_ values are expressed as mean ± SD (*n* = 3–6 independent experiments); ^(1)^ data are in ng/mL; ^(2)^ proportion of polyphenols ca. 40%. EPs^®^ 7630 (root extract of *Pelargonium sidoides*).

**Table 4:** Concentration–effect relationship parameters and mean combination index (CI) values of zanamivir and EPs^®^ 7630 alone and in combinations for the inhibition of the bacterial VCNA.

Compound	Concentration–Effect Parameters		CI Values at				CI_wt_	Combined Effect
	***D_m_***	***m***	**IC_50_**	**IC_75_**	**IC_90_**	**IC_95_**		
zanamivir	17.0 ± 1	0.9	-	-		-	-	-
EPs^®^ 7630	1.7 ± 0.1	1.4	-	-		-	-	-
								
**Ratio of Zanamivir and EPs^®^ 7630**								
5:1	7.8 ± 0.6	1.1	1.1	0.7	0.5	0.4	0.5	synergistic
10:1	9.1 ± 0.4	1.7	0.9	0.7	0.6	0.5	0.6	synergistic
1:1	7.8 ± 0.6	1.7	2.3	1.8	1.5	1.3	1.6	antagonistic
1:5	1.4 ± 0.1	1.2	0.7	0.8	1.0	1.1	1.0	additive
1:10	1.4 ± 0.2	1.0	0.7	0.8	0.8	0.9	0.8	moderate synergistic

The parameters *D_m_* (antilog of the x-intercept) and *m* (slope) are derived from the median effect plot and are used to calculate the CI values (*n* = 3–4 experiments). IC_50_, IC_75_, IC_90_ and IC_95_ are the concentrations (µg/mL) that achieve 50%, 75%, 90% and 95% inhibition of VCNA. The weighted CI (CI_wt_) is calculated on the basis of these representative CI values at effect levels *f_a_* > 0.5.

**Table 5:** Data collection and refinement statistics for determined VCNA-inhibitor complex structures.

	Oseltamivir Carboxylate	Zanamivir
Data Collection		
Space group	*P*2_1_2_1_2_1_	*C*121
Cell dimension		
*a*, *b*, *c* (Å)	71.76, 77.86, 163.48	190.55, 50.34, 86.09
α, β, γ (˚)	90, 90, 90	90, 107.24, 90
Resolution (Å)	44.33–1.87 (1.94–1.87) *	46.00–1.75 (1.81–1.75)
*R*_merge_ (%)	10.1 (49.6)	4.4 (47.8)
<*I*/σ(*I*)>	10.2 (2.4)	14.7 (1.8)
Completeness (%)	99.2 (97.9)	97.8 (96.8)
Multiplicity	3.7 (3.5)	2.3 (2.2)
**Refinement**		
Resolution (Å)	44.33–1.87	46.00–1.75
No. reflections	282260	176727
*R*_work_/*R*_free_ (%)	16.31/20.44	16.69/20.29
No. atoms	6632	6569
Protein	5834	5852
Ligand/ion	42	57
Water	756	660
Average *B*-factor (Å^2^)		
Overall	24.3	32.0
Protein	23.4	31.3
Ligand/ion	22.7	36.0
Water	31.9	38.0
R.m.s. deviations		
Bond lengths (Å)	0.010	0.007
Bond angles (˚)	1.18	1.05

One single-crystal was used to collect a complete dataset for every structure. * Highest resolution shell is shown in parenthesis.

## Supplementary Materials

Supplementary Materials are available online.
