# Supplementary material for: Different Inhibitory Potencies of Oseltamivir Carboxylate, Zanamivir, and Several Tannins on Bacterial and Viral Neuraminidases as Assessed in a Cell-Free Fluorescence-Based Enzyme Inhibition Assay
Source: Molecules. 2017 Nov 17;22(11):1989. doi: 10.3390/molecules22111989 (PMC6150325; doi:10.3390/molecules22111989)
Supplement: Supplementary file 1 [file molecules-22-01989-s001.pdf]

Supplementary Materials

Different Inhibitory Potencies of Oseltamivir Carboxylate, Zanamivir, and Several Tannins on Bacterial and Viral Neuraminidase as Assessed in a Cell-Free Fluorescence-Based Enzyme Inhibition Assay

Stefanie Quosdorf[1], Anja Schuetz[2] and Herbert Kolodziej[1]

[1] Freie Universität Berlin, Institute of Pharmacy, Königin-Luise-Str. 2+4, 14195 Berlin, Germany
[2] Max-Delbrück-Centrum for Molecular Medicine, Helmholtz-Protein Sample Production Facility, Robert-Rössle-Str. 10, 13125 Berlin, Germany

Dedicated to the late Professor emeritus Takuo Okuda

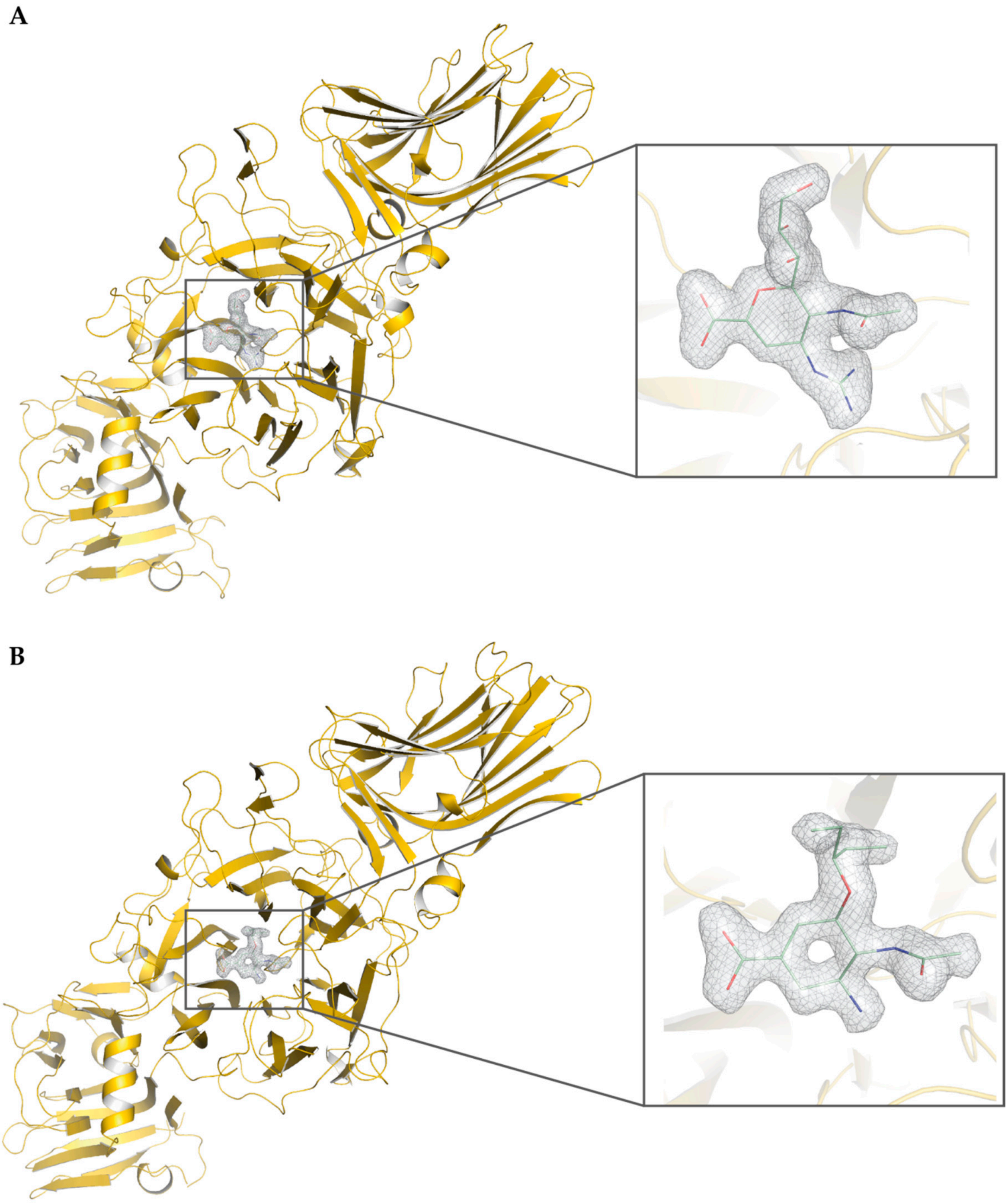

Figure S1. Co-crystal structure of bacterial *Vibrio cholerae* NA in complex with (**A**) zanamivir (PDB code 6EKU, this study) and (**B**) oseltamivir carboxylate (PDB code 6EKS, this study) with enlarged representations of the 2Fo-Fc electron density maps for the bound inhibitors, contoured at 1.0 σ. The bound inhibitor is shown as stick model, VCNA as cartoon model (yellow).

```
H1N1-NA    SVKLAGNSSLCPVSGWAIYSKDNSVRIGSKGDVFVIREPFISC...SPLE   128
VCNA       GSSNTDGVAAYRDIKFEIQGDVIFRGP........DRIPSIVASSVTPGV   237
consensus  ...............f.I..................R.P.I.....tP..

H1N1-NA    CRTFFLTQGALLNDKHSNGTIKDRSPYRTLMSCPIGEVP..........S   168
VCNA       VTAFAEKRVGG.......GDPGALSNTNDIITRTSRDGGITWDTELNLTE   280
consensus  ...F.....g........G.....S....iit....d.............

H1N1-NA    PYNSRFESVAWSASACHDG..INWLTIGISGPDNGAVAVLKYNG......   210
VCNA       QINVSDEFDFSDPRPIYDPSSNTVLVSYARWPTDAAQNGDRIKPWMPNGI   330
consensus  ..N...E..........D......L......P..aA....r.........

H1N1-NA    .ITDTIKSWRNNILRTQESECACV..........NGSCFTVMTDGPSNGQ   250
VCNA       KTGNTMSLYGNASVNPGPGHGITLTRQQNISGSQNGRLIYPAIVLDR.FF   579
consensus  ....Tm..y.N..v.........l..........NG..............

H1N1-NA    ASYKIFRIEKG..KIVKSVEMNAP...........NYHYECSCYPDS.SE   287
VCNA       LNVMSIYSDDGGSNWQTGSTLPIPFRWKSSSILETLEPSEADMVELQNGD   629
consensus  ........d.G.........l..P...............E.........d

H1N1-NA    ITCVCRDNWH.......GSNRPWVSFNQ.NLEYQIGYICSGIFGDNPRPN   329
VCNA       LLLTARLDFNQIVNGVNYSPRQQFLSKDGGITWSLLEANNANVFSNIS..   677
consensus  l....R..f.........S.R.........i.w.l.....a....N....

H1N1-NA    DKTGSCGPVSSNGANGVKGFSFKYG.....NGVWIG....RTKSISSRNG   370
VCNA       .............TGTVDASITRFEQSDGSHFLLFTNPQGNPAGTNGRQN   714
consensus  ................V.a...rf........l..............Rq.

H1N1-NA    FEMIWDPNGWTGTDNNFSIKQDIVGINEWSGYSGSFVQHPELTGLDCIRP   420
VCNA       LGLWFSFDE....GVTWKGPIQLV..NGASAYSDIYQLDS.......ENA   751
consensus  ..l.f..........w......lV..N..SaYS..y..............

H1N1-NA    CFWVELIRGRPKENTIWTSGSSISFCGVNSDTVGWS                 456
VCNA       IVIVETDN..........SNMRILRMPITLLKQKLT                 777
consensus  ...VE.............S...I....i.......t
```

Figure S2. Structure-based amino acid sequence alignment of *Vibrio cholerae* NA (1W0O) and influenza virus H1N1 A/California/04/2009 NA (3NSS). Identical amino acid residues are highlighted in blue, similar amino acid residues in light blue.

Table S1. Concentration–effect relationship parameters and combination index (CI) values of zanamivir and EPs® 7630 alone and in combinations for the inhibition of the bacterial VCNA calculated for exclusivity and non-exclusivity.

compound		CI_{50}	CI_{75}	CI_{90}	CI_{95}	CI_{wt}	combined effect
zanamivir		–	–		–	–	–
EPs® 7630		–	–		–	–	–
ratio of zanamivir/ EPs® 7630							
5:1	exclusive	1.1 ± 0.1	0.7 ± 0.1	0.5 ± 0.1	0.4 ± 0.1	0.5	synergistic
	non-exclusive	1.3 ± 0.1	0.8 ± 0.01	0.5 ± 0.06	0.4 ± 0.07	0.6	synergistic
10:1	exclusive	0.9 ± 0.1	0.7 ± 0.1	0.6 ± 0.1	0.5 ± 0.1	0.6	synergistic
	non-exclusive	1.1 ± 0.1	0.8 ± 0.1	0.6 ± 0.1	0.5 ± 0.1	0.7	moderate synergistic
1:1	exclusive		1.8 ± 0.08	1.5 ± 0.08	1.3 ± 0.08	1.6	antagonistic
	non-exclusive	2.6 ± 0.2	2.1 ± 0.01	1.6 ± 0.02	1.4 ± 0.06	1.7	antagonistic
1:5	exclusive	0.7 ± 0.1	0.8 ± 0.01	1.0 ± 0.1	1.1 ± 0.1	1.0	additive
	non-exclusive	0.7 ± 0.1	0.8 ± 0.1	1.0 ± 0.1	1.2 ± 0.1	1.0	additive
1:10	exclusive	0.7 ± 0.1	0.8 ± 0.1	0.8 ± 0.1	0.9 ± 0.1	0.8	moderate synergistic
	non-exclusive	0.7 ± 0.1	0.8 ± 0.1	0.8 ± 0.1	0.9 ± 0.1	0.8	moderate synergistic

CI values are expressed as mean ± SD (n= 3-6 independent experiments). The weighted CI (CI_{wt}) is calculated on the basis of the representative CI values at effect levels f_a>0.5.

Table S2. Comparison of key viral H1N1-NA–ligand interactions

Protein residue	Ligand group	Distance (Å) zanamivir	Distance (Å) oseltamivir acid	Molecular interactions
Arg 118	2-/1-carboxy-	2.76 / 3.46	2.84 / 3.50	1 ; 4
Glu 119	4-guanidino-/5-amino-	3.22	2.80	2 ; 3
Asp 151	4-guanidino-/5-amino-	2.96/3.01	3.19	2 ; 3
Arg 152	carbonyl- (5-/4-acetamido-)	2.86	2.81	1
Arg 156	4-guanidino-/5-amino-	3.26	-	2
Trp 178	4-guanidino-/5-amino-	2.73/3.11	-	2
Glu 227	4-guanidino-/5-amino-	3.12	-	2 ; 3
Glu 276	6-(1′,2′,3′)-trihydroxypropyl-	2.72 / 2.59	-	2
Arg 292	2-/1-carboxy-	3.19 / 3.24	3.10 / 3.29	1 ; 4
Arg 371	2-/1-carboxy-	2.70 / 2.94	2.64 / 2.83	1 ; 4
Tyr 406	2-/1-carboxy-	-	3.47	1
Tyr 406	ring oxygen	3.14	-	1

Here, we analyzed the published co-crystal structures of H1N1-NA in complex with zanamivir and oseltamivir carboxylate (PDB ID codes 3TI5 and 3TI6). Hydrogen bond distances are indicated as derived from COOT analysis. Type of interaction: 1) hydrogen bond (acceptor), 2) hydrogen bond (donor), 3) positive ionization, 4) negative ionization.

Table S3. Comparison of key bacterial VCNA–ligand interactions.

Protein residue	Ligand group	Distance (Å) zanamivir	Distance (Å) oseltamivir acid	Molecular interactions
Arg 224	2-/1-carboxy-	2.89 / 3.04	2.77 / 3.27	1 ; 4
Glu 243	4-guanidino-/5-amino-	2.77 / 3.33	-	2 ; 3
Arg 245	4-guanidino-/5-amino-	2.87 / 3.43	3.39	2
Asp 250	4-guanidino-/5-amino-	2.97 / 3.51	2.97	2 ; 3
Asp 292	4-guanidino-/5-amino-	3.11 / 3.41	-	2 ; 3
Asn 318	carbonyl- (5-/4-acetamido-)	3.37 / 3.54	3.59	1
Asn 318	6-(1′,2′,3′)-trihydroxypropyl-	2.70 / 3.47	-	2
Arg 635	2-/1-carboxy-	2.97 / 3.31	3.00 / 3.19	1 ; 4
Asp 637	6-(1′,2′,3′)-trihydroxypropyl-	2.71 / 3.12	-	2
Arg 712	2-/1-carboxy-	2.85/ 2.87	2.74 / 2.93	1 ; 4
Tyr 740	ring system	3.18	-	1

Here, we analyzed the co-crystal structures of bacterial VCNA in complex with zanamivir and oseltamivir carboxylate, determined in this study (PDB ID codes 6EKU and 6EKS). Hydrogen bond distances are indicated as derived from COOT analysis. Type of interaction: 1) hydrogen bond (acceptor), 2) hydrogen bond (donor), 3) positive ionization, 4) negative ionization.
